# Identification of a novel chromosome-encoded fosfomycin resistance gene *fosC3* in *Aeromonas caviae*

**DOI:** 10.3389/fmicb.2025.1577167

**Published:** 2025-04-15

**Authors:** Junwan Lu, Runzhi Zhang, Yan Yu, Hongqiang Lou, Dong Li, Qiyu Bao, Chunlin Feng

**Affiliations:** ^1^Medical Molecular Biology Laboratory, School of Medicine, Jinhua University of Vocational Technology, Jinhua, China; ^2^Key Laboratory of Medical Genetics of Zhejiang Province, Key Laboratory of Laboratory Medicine, Ministry of Education, School of Laboratory Medicine and Life Sciences, Institute of Biomedical Informatics, Wenzhou Medical University, Wenzhou, China

**Keywords:** fosfomycin, *Aeromonas caviae*, FosC3, enzyme kinetics, antibiotic resistance

## Abstract

**Background:**

Owing to the rapid emerging of multidrug-, even pandrug-resistant pathogens, and lack of new antibiotics, the older antibiotic, fosfomycin, has been reused in recent years in the clinical practice, especially for treatment of uropathogen infections. With the increased use of fosfomycin, bacterial resistance to it has also increased drastically. Elucidating the resistance mechanism to the antimicrobial has become an urgent task.

**Methods:**

The putative fosfomycin resistance gene *fosC3* was cloned, and minimal inhibitory concentrations were determined by the agar dilution method. Enzyme kinetic parameters were measured by high-performance liquid chromatography. Bioinformatics analysis was applied to understand the evolutionary characteristics of FosC3.

**Results:**

The *A. caviae* strain DW0021 exhibited high level resistance to several antimicrobials including kanamycin, streptomycin, chloramphenicol, florfenicol, tetracycline, and especially higher to fosfomycin (> 1,024 μg/mL), while genome annotation indicated that no function-characterized resistance gene was associated with fosfomycin resistance. A novel functional gene designated *fosC3* responsible for fosfomycin resistance was identified in the chromosome of *A. caviae* DW0021. Among the function-characterized proteins, FosC3 shared the highest amino acid similarity of 58.65% with FosC2. No mobile genetic element (MGE) was found surrounding the *fosC3* gene. The recombinant pMD19-*fosC3*/DH5α displayed a MIC value of 32 μg/mL to fosfomycin, which revealed a 128-fold increase of MIC value to fosfomycin compared to the control pMD19/*E. coli* DH5α (0.25 μg/mL). FosC3 was phylogenetically close to FosC2 and exhibited a *k*_cat_ and *K*_m_ of 82,442 ± 1,475 s^−1^, 70.99 ± 4.31 μM, respectively, and a catalytic efficiency of (1.2 ± 0.3) × 10^3^ μM^−1^·s^−1^.

**Conclusion:**

In this work, a novel functional fosfomycin thiol transferase, FosC3, which shared the highest protein sequence similarity with FosC2, was identified in *A. caviae*. The fosfomycin inactivation enzyme FosC3 could effectively inactivate fosfomycin by chemical modification. It is implied that such mechanism facilitates *A. caviae* to respond to fosfomycin exposure, thereby enhancing survival. However, *fosC3* was not related with any MGE, which differs from many other fosfomycin thiol transferase genes. As a result, *fosC3* is not expected to be transmitted to other species through horizontal gene transfer mechanism. Our findings will contribute to the resistance mechanism of the common pathogenic *A. caviae*.

## Introduction

Classified under the Gammaproteobacteria, order Aeromonadales, and family Aeromonadaceae, *Aeromonas* spp. are Gram-negative facultative anaerobes and isolated from numerous sources such as animals, water, soil, and food. As a result, both immunocompromised and immunocompetent humans are more likely to be infected by *Aeromonas* (Pessoa et al., 2022). Common diseases are gastroenteritis, bacteremia, septicemia, etc. (Fernández-Bravo and Figueras, 2020).

The wastewater discharged from hospitals and aquaculture farms is a significant source of multidrug-resistant pathogens and *Aeromonas* spp. in these aquatic environments exhibit a broad spectrum of antibiotic resistance profile (Pessoa et al., 2022). In addition, antibiotic resistance genes could be transmitted between *Aeromonas* via mobile genetic elements such as integrons and plasmids (Bello-López et al., 2019).

The resistance to fosfomycin tends to rise in the settings with use of more fosfomycin and among multi-drug resistant pathogens (Falagas et al., 2019). Three major fosfomycin resistance mechanisms are as follows: (i) reduced permeability to fosfomycin in consequence of mutated fosfomycin intake genes (*uhpT* etc.), (ii) mutations in the fosfomycin target gene *murA*, which takes part in the biosynthesis of peptidoglycan, and (iii) fosfomycin-inactivating enzymes (FosA etc.) (Zurfluh et al., 2020). Fosfomycin-inactivating enzymes are generally associated with mobile genetic elements and therefore have a critical impact in the horizontal transfer of fosfomycin resistance (Güneri et al., 2022). Antibiotic modification by fosfomycin-modifying enzymes is one of the acquired resistance mechanisms (Falagas et al., 2019). Inactivation of fosfomycin could be achieved through (i) addition of the sulphydryl group to C1 of the epoxide ring in fosfomycin (FosA and FosC2); (ii) nucleophilic addition of l-Cys or bacillithiol to fosfomycin (FosB); (iii) addition of H_2_O to the C1 position of epoxide ring in fosfomycin (FosX); (iv) phosphorylation of the phosphate group to monophosphate (FomA and FosC) and conversion of monophosphate to diphosphate (FomB) in fosfomycin (Falagas et al., 2019).

Up to date, at least 10 types of *fos* genes have been discovered, e.g., *fosA*, *fosB*, *fosC,* and so on (Yang et al., 2019). *fosC2* is mainly responsible for fosfomycin resistance in Enterobacteriaceae (Yang et al., 2019). In this work, we identified and characterized a novel chromosome-encoded fosfomycin resistance gene designated *fosC3* from a *A. caviae* strain DW0021.

## Materials and methods

### Bacterial strains and plasmids

The bacteria and plasmids used in this work were listed in Table 1. DW0021 was isolated from a soil sample at an animal farm in Wenzhou, China. The taxonomic classification analysis was a combination of 16S rRNA gene homology and whole-genome average nucleotide identity (ANI). The recommended 95% threshold of ANI was used for species delimitation (Richter and Rosselló-Móra, 2009).

**Table 1:** Bacteria and plasmids used in this study.

Strain and plasmid	Description	References
DW0021	The wild-type strain of *A. caviae* DW0021	This study
DH5α	*E. coli* DH5α as a host for cloning of the *fosC3* gene	Our laboratory collection
BL21	*E. coli* BL21 as a host for expression of the *fosC3* gene	Our laboratory collection
ATCC 25922	*E. coli* ATCC 25922 as the quality control for antimicrobial susceptibility testing	Our laboratory collection
pMD19-*fosC3*/DH5α	DH5α carrying the recombinant plasmid pMD19-*fosC3*	This study
pCold I-*fosC3*/BL21	BL21 carrying the recombinant plasmid pCold I-*fosC3*	This study
pMD19	Cloning vector for the *fosC3* gene with its upstream promoter region, AMP^r^	Our laboratory collection
pMD19-*fosC3*	A recombinant plasmid of pMD19 carrying the *fosC3* gene with its upstream promoter region	This study
pCold I	Expression vector for the ORF of the *fosC3* gene, AMP^r^	Our laboratory collection
pCold I-*fosC3*	A recombinant plasmid of pCold I carrying the ORF of the *fosC3* gene	This study

AMP^r^, Ampicillin resistance.

### Minimum inhibitory concentration determination

The antimicrobial agents used in antimicrobial susceptibility testing were in Table 2, which included streptomycin, kanamycin, chloramphenicol, florfenicol, tetracycline, fosfomycin and so on. The antimicrobial susceptibility testing was performed following the antimicrobial susceptibility testing standard M100 (34th Edition, 2024) from the Clinical and Laboratory Standards Institute.

**Table 2:** MICs (μg/mL) of *A. caviae* DW0021, the cloned *fosC3* gene and the controls.

Antibiotics	DH5α	pMD19/DH5α	*E. coli* ATCC 25922	pMD19-*fosC3*/DH5α	*A. caviae* DW0021^*^	Genes
Tobramycin	2	2	2	4	128 ^R^	*aph(3″)-Ib, aph(6)-Id, aac(6′)-Ib9 × 2*
Streptomycin	8	8	8	16	128^NA^	
Kanamycin	4	4	4	4	512^R^	
Chloramphenicol	8	8	8	16	128 ^R^	*cmlA5, floR*
Florfenicol	8	8	8	16	64 ^NA^	
Tetracycline	4	4	4	4	128 ^R^	*tet(E), tet(A)*
Fosfomycin	0.25	0.25	0.25	32	>1,024 ^R^	*fosC3*
Ceftazidime	2	4	2	4	64 ^R^	*bla_OXA-10_, bla_OXA-504_, bla_MOX-3_*
Cefotaxime	2	2	2	2	2 ^S^	
Aztreonam	1	1	1	1	1 ^S^	
Meropenem	1	1	1	1	1 ^S^	
Levofloxacin	1	1	0.5	1	32 ^R^	*qnrVC4*
Sulfonamides	2	2	4	4	512 ^R^	*sul1*

* Interpretive criteria were adapted from CLSI document M100 for Enterobacteriaceae. NA, Interpretive criteria not available; S, Sensitive; R, Resistant.

### Molecular cloning of the *fosC3* gene

Primers were designed using Primer Premier^1^ (PREMIER Biosoft International, Palo Alto, CA) and SnapGene^2^ (Table 3). The open reading frame (ORF) of *fosC3* with the promoter region was amplified and inserted into the T-Vector pMD19 with the T4 DNA ligase (Takara Bio, Inc., Dalian, China). The constructed recombinant plasmid was transformed into *E. coli* DH5α using the calcium chloride method and cultured on Luria-Bertani agar plates containing 100 μg/mL ampicillin. The inserted sequences were verified by sequencing (Shanghai Sunny Biotechnology Co., Ltd., Shanghai, China).

**Table 3:** PCR profiles for the amplification of *fosC3* gene.

Primer^1^	Sequence (5′ → 3′)^2^	Restriction endonuclease	Vector	Annealing temperature (°C)	Amplicon size (bp)
pro-*fosC3*-F	ATCGATGGGGTTGACCCCCGCAAAG		pMD19	67	841
pro-*fosC3*-R	TGGGGCAGGGCAGCAATCGTCACTAC		pMD19		841
orf-*fosC3*-F	CCGGAATTCCTGGTGCCGCGCGGCAGCATGCTGCATGGACTGAACCATCTCAC	*Eco*RI*+* Thrombin	pCold I	63	438
orf-*fosC3*-R	TGCTCTAGATCAGTCGAACCAGATAAGACCCTCATAGGG	XbaI	pCold I		438

1 Primers started with “pro” were used to clone the *fosC3* gene with its promoter region; primers started with “orf” were used to clone the ORF of the *fosC3* gene.

2 The underlined sequences indicated the restriction endonuclease sites.

### Expression and purification of the FosC3 enzyme

The ORF of *fosC3* was PCR-amplified and inserted into the pCold I vector with the cleavage sites of thrombin, restriction endonucleases *Eco*RI and *Xba*I (Table 3). The recombinant plasmid was transformed into *E. coli* BL21. FosC3 was overexpressed and purified as described previously (Qing et al., 2004). After the OD_600_ reached 0.6 at 37°C, FosC3 enzyme induction occurred in the presence of 0.1 mM isopropyl-*β*-d-thiogalactoside, followed by 20 h cultivation at 16°C. Bacteria were centrifugated (5,000 × g, 10 min) at 4°C, resuspended in lysis buffer (20 mM Tris–HCl, 150 mM NaCl, 3 mM β-mercaptoethanol, 0.5% Nonidet-P-40, pH 8.0), and disintegrated by sonication. The cell debris was eliminated through centrifugation (12,000 × g, 30 min) at 4°C. Subsequently, the lysates were incubated with pre-equilibrated nickel-nitrilotriacetic acid (Ni-NTA) agarose resin (Beyotime Biotechnology, Shanghai, China) for 8 h at 4°C under slow agitation. The recombinant protein purification was achieved using standard Ni-NTA affinity chromatography. The His6 tag was removed by incubation with thrombin for 4 h at 37°C. The concentration of the purified FosC3 protein was measured by SDS-PAGE and the BCA protein assay kit (Thermo Fisher Scientific, Rockford, IL, United States).

### Enzyme kinetic parameter determination of the FosC3 enzyme

The kinetic assay was based on high-performance liquid chromatography (HPLC) and the methods described previously (Rigsby et al., 2005). The components of reaction mixture were listed in Table 4. After 5 min of incubation at 37°C, the reaction was terminated by 900 μL solution contain 90 and 10% volume of mobile phase A (100 mM KH_2_PO_3_-H_3_PO_3_) and B (methanol), respectively. Then the mixture was centrifugated at 12,000 rpm for 10 min. 700 μL supernatant was injected into a 250 mm × 4.6 mm Elite C-18 column (GL Sciences, Shanghai, China) with a flow rate of 800 μL/min. Analysis was done by the Accela UHPLC system (Thermo Fisher Scientific, Rockford, IL, United States) under the 200 nm wavelength.

**Table 4:** Components of reaction mixture for enzyme kinetic parameter determination.

Component	Concentration	Volume (μL)
H_2_O	/	48
MnCl_2_	2.5 mM	2
Tris–HCl	1 M	10
KCl	1 M	10
GSH	10 mM	10
Enzyme	0.015 mg/mL	10
Fosfomycin	concentration gradients^a^	10

a 250, 500, 1,000, 1,500, 2,000, 3,000, 4,000, 6,000 μM.

### Whole genome sequencing, genome assembly, and annotation

The whole-genome sequencing was processed on the Illumina NovaSeq (paired-end, 2 × 150 bp) and PacBio RS II (20 kbp library) platforms (Shanghai Personal Biotechnology Co., Ltd., Shanghai, China), respectively. The genome sequence was obtained through Unicycler assembly pipeline (Wick et al., 2017). The long reads were assembled by miniasm (Li, 2016) and the genome was polished with Illumina short reads by Racon (Vaser et al., 2017). Protein-, tRNA-and rRNA-coding sequences (CDSs) were found by Prodigal (Hyatt et al., 2010), ARAGORN (Laslett and Canback, 2004) and Barrnap^3^, respectively. The promoter region was predicated by BPROM (Solovyev, 2011). The annotation of protein sequences was based on alignment of the predicted CDSs to the NCBI nr database (Sayers et al., 2021), the Swiss-Prot database (Bateman et al., 2023), and the Comprehensive Antibiotic Resistance Database (CARD) (Alcock et al., 2022) using DIAMOND blastp (Buchfink et al., 2021). The genome of DW0021 was served as the reference genome and the other 9 *A. caviae* genomes sharing the highest nucleotide identity with DW0021 were used for comparison (Table 5). Comparison of different *A. caviae* genomes was visualized by CGView Comparison Tool (Grant et al., 2012). The molecular weight and isoelectric point (pI) of protein sequences were calculated by EMBOSS pepstats (Rice et al., 2000).

**Table 5:** Comparison of the 9 genomes of *A. caviae*.

Bacterium	Accession	Size (bp)	CDSs	tRNA	rRNA (5S, 16S, 23S)	Resistance genes	Virulence factors	Plasmids
DW0021	CP128475.1	4,589,869	4,086	126	11, 10, 10	11	56	1
FAHZZU2447	CP100392.1	4,540,521	4,254	124	11, 10, 10	11	59	2
NCTC12244	LS483441.1	4,586,140	4,048	123	11, 10, 10	2	76	0
FDAARGOS_72	CP062787.1	4,527,600	3,741	122	11, 10, 10	2	73	0
FDAARGOS_75	CP062801.1	4,551,146	3,871	122	11, 10, 10	2	73	0
211,703	CP092181.1	4,783,384	4,270	124	11, 10, 10	14	76	0
KAM376	AP024402.1	4,664,715	4,441	120	11, 10, 10	20	58	7
71,442	CP084350.1	4,444,683	3,922	122	11, 10, 10	7	41	0
R25-6	CP025705.1	4,702,913	4,146	123	11, 10, 10	11	48	1
NUITM-VA2	AP025280.1	5,035,951	4,473	123	11, 10, 10	31	41	0

### Phylogenetic tree reconstruction and model building

Entrez Direct^4^ and GNU Parallel (Tange, 2021) were used to retrieve sequences from the NCBI databases. Samtools (Danecek et al., 2021) and SeqKit (Shen et al., 2016) were used to manipulate fasta sequences. FosC3 and the other sequences were aligned with L-INS-i strategy by MAFFT (Katoh and Standley, 2013) and printed by r-msa (Bodenhofer et al., 2015). The maximum-likelihood tree of FosC3 and its homologous sequences was reconstructed with LG + G4 substitution model, tested by 1,000 bootstrap replicates and visualized by IQ-TREE 2 (Minh et al., 2020), UFBoot2 (Hoang et al., 2018) and ggtree (Yu, 2020), respectively. The structure of FosC3 and FosC3 bound fosfomycin was modeled by AlphaFold 3 and SWISS-MODEL using the fosfomycin resistance protein with bound fosfomycin (SMTL ID: 5v3d.1) as template (Waterhouse et al., 2018; Abramson et al., 2024).

### Gene synteny analysis

The *fosC3* gene, along with its flanking regions, were used as queries and searched against the NCBI non-redundant nucleotide database to infer synteny. Comparison of gene clusters was generated by clinker (Gilchrist and Chooi, 2021).

### Nucleotide sequence accession numbers

The GenBank accession numbers of the sequences of the *A. caviae* DW0021 chromosome, plasmid pAECA21-3829 and *fosC3* gene were CP128475, CP128476, and OR187734, respectively.

## Results and discussion

### Antibiotic resistance pattern and genomic analysis of DW0021

Among 13 antimicrobials tested, *A. caviae* DW0021 showed high MIC values (≥ 32 μg/mL) to 10 of them, including kanamycin (512 μg/mL), tobramycin (128 μg/mL), streptomycin (128 μg/mL), chloramphenicol (128 μg/mL), florfenicol (64 μg/mL), tetracycline (128 μg/mL), and especially higher to fosfomycin (>1,024 μg/mL) (Table 2).

To analyze the molecular resistance mechanism, the complete genome of *A. caviae* DW0021 was sequenced, and it consisted of a chromosome and a plasmid designated pAECA21-3829. The chromosome was 4,589,869 bp in length with 61.43% GC content, encoding 4,086 proteins, 126 tRNAs and 31 rRNAs. pAECA21-3829 was 3,829 bp in length, harboring 6 CDSs (Table 6). The 16S rRNA gene sequence analysis indicated that DW0021 was phylogenetically closer to *Aeromonas* spp., and genome wide sequence comparison of DW0021 with those genomes available in the NCBI genome database revealed that it shared the highest whole-genome ANI (97.99%) with the *A. caviae* type strain NCTC12244 (genome assembly accession number: GCA_900476005.1). Therefore, the strain DW0021 was classified into *A. caviae* and named *A. caviae* DW0021. The annotation result of antimicrobial resistance genes was listed in Table 7. Thirteen antimicrobial resistance genes (≥ 80% similarity with the function-characterized resistance genes in the CARD database) that related to six classes of antimicrobial agents were annotated, including aminoglycoside [*aph(3″)-Ib*, *aph(6)-Id*, and two copies of *aac(6′)-Ib9*], *β*-lactam (*bla*_OXA-10_, *bla*_OXA-504_, and *bla*_MOX-3_), fluoroquinolone (*qnrVC4*), phenicol (*cmlA5* and *floR*), sulfonamide (*sul1*), and tetracycline [*tet(E)* and *tet(A)*]. All the antimicrobial resistance genes were encoded on the chromosome, while the plasmid pAECA21-3829 was free of an antimicrobial resistance gene.

**Table 6:** Genome features of *A. caviae* DW0021.

Property	Chromosome	Plasmid (pAECA21-3829)
Accession number	CP128475	CP128476
Size (bp)	4,589,869	3,829
GC content (%)	61.43	55.68
CDS	4,086	6
Known protein	2,868	0
Hypothetical protein	1,218	6
Average protein length	322	113
tRNA	126	0
rRNA	31	0

**Table 7:** Annotation of antimicrobial resistance genes in *A. caviae* DW0021.

Locus	Coverage (%)	Identity (%)	E-value	Gene	Related antimicrobial class
DW0021-chr_00343	100.0	99.6	4.72E-202	*aph(3″)-Ib*	Aminoglycoside
DW0021-chr_00344	100.0	99.0	1.81E-150	*aph(6)-Id*	Aminoglycoside
DW0021-chr_00347	95.5	99.5	5.08E-142	*aac(6′)-Ib9*	Aminoglycoside
DW0021-chr_00350	99.0	100.0	2.23E-144	*aac(6′)-Ib9*	Aminoglycoside
DW0021-chr_00349	100.0	100.0	1.76E-192	*bla_OXA-10_*	β-lactam
DW0021-chr_04216	100.0	97.3	3.71E-193	*bla_OXA-504_*	β-lactam
DW0021-chr_02969	100.0	95.3	2.25E-267	*bla_MOX-3_*	β-lactam
DW0021-chr_00346	100.0	100.0	7.46E-80	*qnrVC4*	Fluoroquinolone
DW0021-chr_00348	100.0	100.0	8.39E-300	*cmlA5*	Phenicol
DW0021-chr_00357	100.0	99.3	1.04E-274	*floR*	Phenicol
DW0021-chr_00366	100.0	100.0	2.32E-199	*sul1*	Sulfonamide
DW0021-chr_00333	100.0	99.8	1.03E-286	*tet(E)*	Tetracycline
DW0021-chr_00354	92.8	98.7	1.24E-160	*tet(A)*	Tetracycline

**Figure 1:**
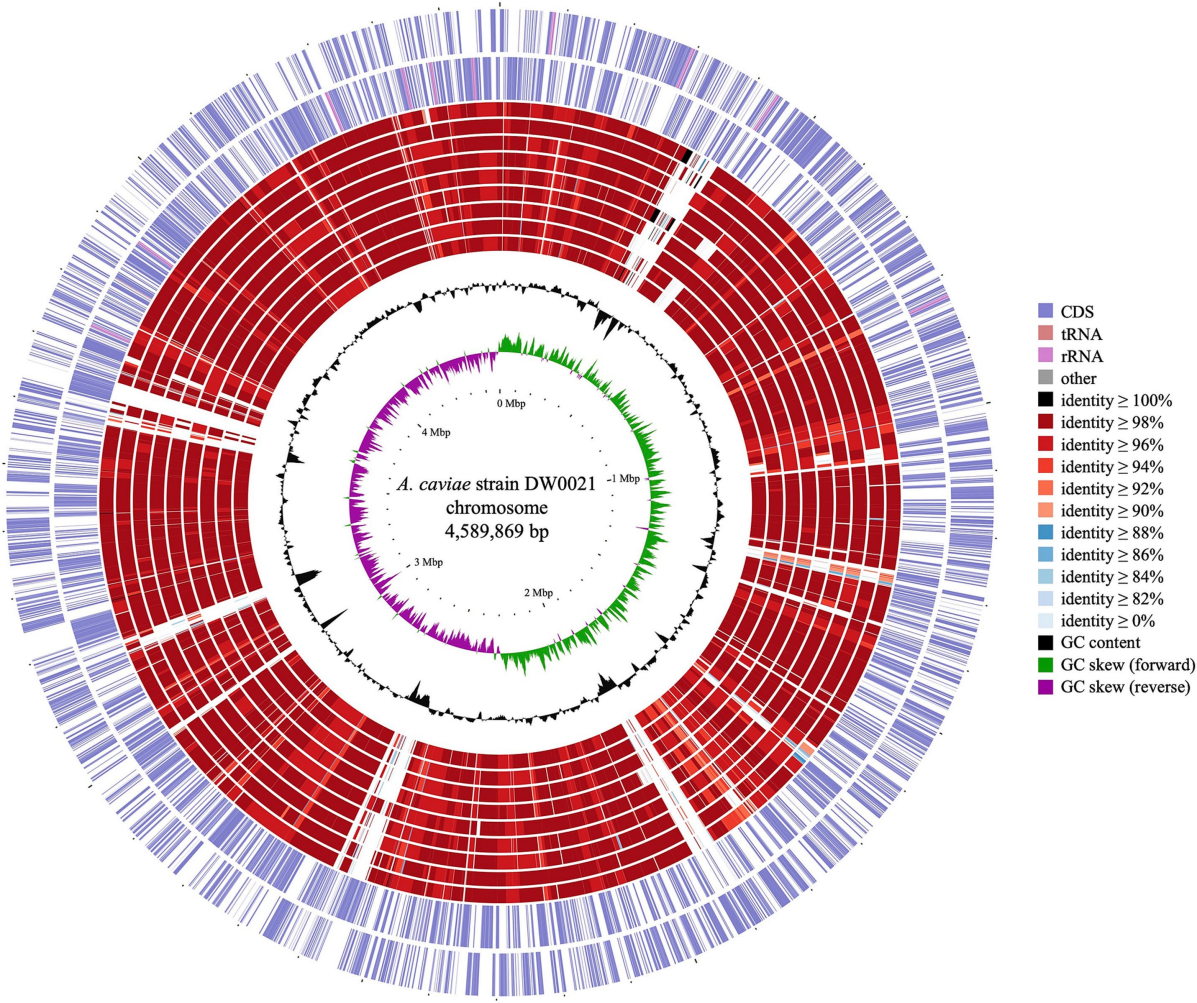
Genome map of *Aeromonas caviae* DW0021 and other *A. caviae* strains. Circles (from outside to inside) 1–2, indicated forward and reverse strand of the DW0021 chromosome (CP128475.1); 3–11 were the chromosomes of *A. caviae* KAM376 (AP024402.1), *A. caviae* FAHZZU2447 (CP100392.1), *A. caviae* FDAARGOS_75 (CP062801.1), *A. caviae* FDAARGOS_72 (CP062787.1), *A. caviae* NUITM-VA2 (AP025280.1), *A. caviae* 211703 (CP092181.1), *A. caviae* NCTC12244 (LS483441.1), *A. caviae* 71442 (CP084350.1), and *A. caviae* R25-6 (CP025705.1), respectively; 12–13 were GC content and GC skew of the DW0021 chromosome, respectively.

Comparative genomic analysis of DW0021 and the other *A. caviae* strains was shown in Figure 1. It revealed that the genome of *A. caviae* DW0021 was similar to the other 9 *A. caviae* genomes in large part.

### *fosC3* showing resistance to fosfomycin

Although *A. caviae* DW0021 exhibited high level MIC to fosfomycin, the 13 predicted resistance genes from the whole genome were not associated with the resistance to the antimicrobial. A novel fosfomycin resistance mechanism would be present in *A. caviae* DW0021, which may be affiliated with an unidentified antimicrobial resistance gene. To confirm the speculation, the annotation result of the genome sequence was examined and the deduced protein sequence encoded by one *fosC2*-like gene sharing 99.25% coverage and 59.09% identity with FosC2 (BAJ10053.1) was found. The *fosC2*-like gene (designated *fosC3* in this work) was cloned and confirmed to be functional. The recombinant pMD19-*fosC3*/DH5α displayed a 128-fold increase of MIC value to fosfomycin (32 μg/mL) compared to pMD19/DH5α (0.25 μg/mL), however, no significantly reduced susceptibility to the other antimicrobial agents identified (Table 2).

Generally, the bacteria with the fosfomycin-modifying genes (*fos* and *fom* genes) showed high MIC levels to fosfomycin. For example, *P. syringae* PB-5123 carrying a *fosC* gene demonstrated a MIC of 1,024 mg/mL to fosfomycin (García et al., 1995). Compared to the recipients, the transformant DH10B (pS-fosC2) with *fosC2* showed reduced susceptibility (MIC > 256 μg/mL, increased > 512-fold) (Wachino et al., 2010), and the FosC2^AS^-producing *E. coli* recombinant also showed high level of resistance to fosfomycin (MIC > 256 μg/mL, increased > 64-fold) (Ortiz de la Rosa et al., 2022). Other reports also revealed high or increased MIC levels when a strain harbored a fosfomycin resistance gene or a mutated one. *E. coli* KAM32/pSP72/Vf-*murA* had an MIC of 3,000 μg/mL to fosfomycin (Kumar et al., 2009). Amino acid substituted MurA found in resistant isolates further raised the MIC for fosfomycin by more than 8-fold (≥ 1,024 mg/L) compared with the strains expressing wild-type MurA (Takahata et al., 2010). The fosfomycin-resistant subpopulations overexpressed *murA* also resulted in a 10-fold increase (from 0.064 to 0.64 mg/L) (Campos da Campos et al., 2020). Another gene called *abrp* conferred a 4-fold decreased susceptibility to fosfomycin (from 64 mg/L to 256 mg/L) in *A. baumannii* (Li et al., 2016). Mutations in the structure of *glpT* and *uhpT* showed increased MICs to fosfomycin (≥ 256 mg/L) (Takahata et al., 2010).

The 402 bp ORF of *fosC3* gene encoded a 133 amino acid enzyme with a molecular weight of 14.87 kDa and pI of 5.96. To further study the properties of the novel enzyme, FosC3 was over-expressed (Supplementary Figure S1A) and purified (Supplementary Figure S1B). Enzyme kinetic assays of FosC3 based on HPLC (Supplementary Figure S2) manifested a *k*_cat_ and *K*_m_ of 82,442 ± 1,475 s^−1^, 70.99 ± 4.31 μM, respectively, which indicated that FosC3 could inactivate fosfomycin with a catalytic efficiency (*k*_cat_/*K*_m_) of (1.2 ± 0.3) × 10^3^ μM^−1^·s^−1^. Most kinetic analyses of fosfomycin thiol transferases were conducted on FosAs (Table 8). FosA typically demonstrated a high catalytic efficiency (≥ 10^3^), which may be a result of selection pressures exerted by the clinical use of fosfomycin. Although the *k*_cat_ of FosC3 (82,442 ± 1,475 s^−1^) was significantly more than that of other fosfomycin thiol transferases, no notable difference of catalytic efficiency was observed. The results indicate that the FosC3 was probably less or equal active than the FosA enzyme variants.

**Table 8:** Enzyme kinetics of FosC3 and other fosfomycin-inactivating enzymes.

Enzyme	*k*_cat_ (s^−1^)	*k*_cat_/*K*_m_ (M^−1^ s^−1^)	References
FosC3	82,442 ± 1,475	(1.2 ± 0.3) × 10^3^	This study
FosA3	112.4 ± 5.3	(9.0 ± 1.0) × 10^3^	Lu et al. (2024)
FosA11	56.1 ± 3.2	(2.9 ± 0.5) × 10^3^	
FosX	34 ± 2	(9.0 ± 2.0) × 10^4^	Fillgrove et al. (2007)
FosX	0.15 ± 0.02	(5.0 ± 0.6) × 10^2^	Fillgrove et al. (2003)
FosB	NA^a^	(1.7 ± 0.3) × 10^5^	Cao et al. (2001)
FosA	180 ± 6	(4.1 ± 0.8) × 10^4^	Rigsby et al. (2007)
FosA	80 ± 2	(2.1 ± 0.1) × 10^5^	Rife et al. (2002)
FosA	180 ± 6	(9.0 ± 1.4) × 10^5^	Brown et al. (2009)
FosA	42.1 ± 4.5	(3.7 ± 1.0) × 10^3^	Klontz et al. (2017)
FosA3	99.4 ± 3.3	(8.0 ± 1.9) × 10^3^	
FosA	140 ± 15	(1.0 ± 1.3) × 10^4^	

^aNot available.^

### Comparative analysis of *fosC3* with other *fos* genes

**Figure 2:**
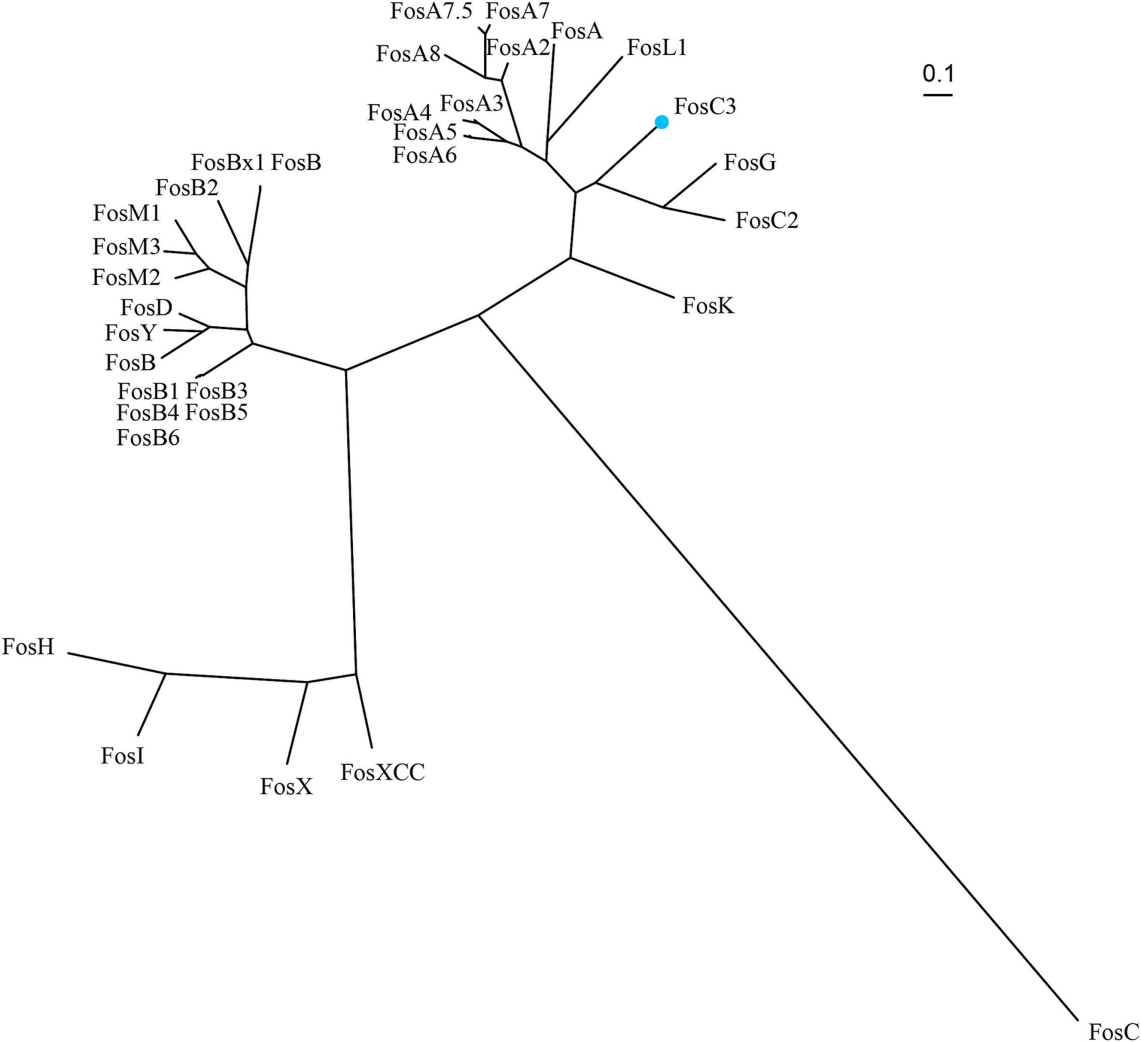
Unrooted phylogenetic tree of FosC3 and other function-characterized fosfomycin-modifying enzymes. FosC3 (blue dot) was in the clade that close to FosG and FosC2. Accession numbers: FosC3 (XBY83663.1), FosA (AAG04518.1), FosA2 (ACC85616.1), FosA3 (AEG78825.1), FosA4 (BAP18892.1), fosA5 (AJE60855.1), FosA6 (AMQ12811.1), FosA7 (KKE03230.1), FosA7.5 (ANQ03635.1), FosA8 (QEI22965.1), FosB (AAP08996.1), FosB1 (BAE05988.1), FosB2 (AAP27834.1), FosB3 (ADX95999.1), FosB4 (ALM24139.1), FosB5 (ALN12426.1), FosB6 (ALM24145.1), FosBx1 (QLF01382.1), FosC (CAA83855.1), FosC2 (BAJ10053.1), FosD (BAG12271.1), FosG (RTB44598.1), FosH (ADF48907.1), FosI (AFJ38137.1), FosK (BAO79518.1), FosL1 (QHR93773.1), FosM1 (DAC85639.1), FosM2 (DAC85640.1), FosM3 (DAC85641.1), FosX (CWV56762.1), FosXCC (AIF29598.1), FosY (QTE33800.1), and FosB (EHS19134.1).

*fosC3* was a novel fosfomycin-modifying gene. The phylogenetic tree of FosC3 and the other function-characterized fosfomycin-modifying enzymes was depicted in Figure 2. In the phylogenetic tree, FosC3 was on a branch that was close to FosC2 and FosG. Among the function-characterized Fos proteins, FosC3 shared the highest amino acid similarity of 58.65% (99.25% coverage and 59.09% identity) with FosC2. FosC3 also shared > 50% similarities with FosG, FosK, FosA5, FosA6, FosA, FosL1, FosA8, FosA2, FosA3, FosA7.5, FosA4, and FosA7, but < 40% with the rest fosfomycin thiol transferases.

**Figure 3:**
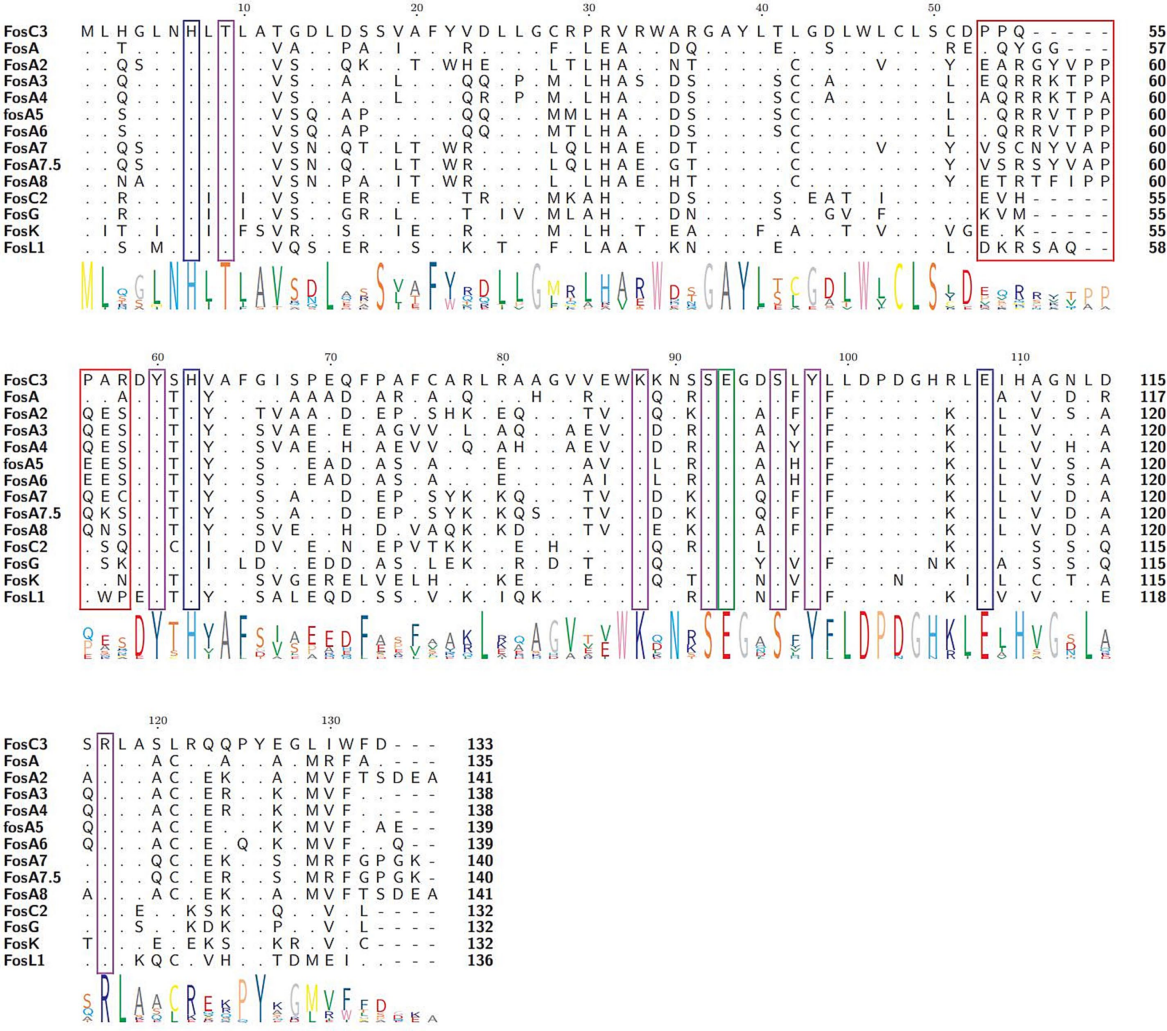
Multiple sequence alignment of FosC3 and its homologous fosfomycin-modifying enzymes. The length of each sequence was labeled on the right. The logo size at the bottom indicated the degree of conservation at that residue. Dots and hyphens represented identical residues and gaps, respectively. Amino acid residues in the red, blue, green, and purple frames manifested the dimer interface loop of enzymes, with residues involved in Mn^2+^ coordination, K^+^ binding loop, and fosfomycin binding, respectively. Accession numbers: FosC3 (XBY83663.1), FosA (AAG04518.1), FosA2 (ACC85616.1), FosA3 (AEG78825.1), FosA4 (BAP18892.1), fosA5 (AJE60855.1), FosA6 (AMQ12811.1), FosA7 (KKE03230.1), FosA7.5 (ANQ03635.1), FosA8 (QEI22965.1), FosC2 (BAJ10053.1), FosG (RTB44598.1), FosK (BAO79518.1), and FosL1 (QHR93773.1).

FosC3 possessed the similar functional residues that were similar to FosA. Although FosA and FosC2 were two distinct enzymes, both could inactivate fosfomycin through glutathione *S*-transferase activity (Wachino et al., 2010). FosA proteins possessed residues that were responsible for the dimer interface loop (Figure 3, amino acid residues 53 to 58 in red frames), Mn^2+^ (residues 7H, 62H, 108E, blue frames) and K^+^ binding (93E, green frames), and fosfomycin binding (9 T, 60Y, 88 K, 92S, 96S, 98Y, purple frames) (Klontz et al., 2017). Multiple sequence alignment of FosC3 and its homologous fosfomycin-modifying enzymes revealed that FosC3 contained the similar residues (Figure 3) and may be able to inactivate fosfomycin through glutathione *S*-transferase activity (Wachino et al., 2010). The notable divergence was related to the dimer interface loop among fosfomycin thiol transferases, while those residues involved in Mn^2+^ coordination, K^+^ binding, and fosfomycin binding were identical. FosC3, FosC2, FosG and FosK were five residues shorter, and that of FosA and FosL1 were three and two, which implied that the loop could cross the dimer interface relatively directly (Klontz et al., 2017). The enzyme was predicted to a homo-dimer (Supplementary Figure S3) with residues 9T, 46W, 60Y, 88K, 92S, 98Y, 117R to bind with fosfomycin. FosC3 was also predicted to form metal complexes with K^+^ (90N, 92S, 94G, 96S) and Mn^2+^ (7H, 62H, 108E).

*Aeromonas* spp. could be a reservoir of *fosC3* genes. It was found that 54 FosC3-like sequences (annotated as putative fosfomycin-modifying enzymes) sharing > 90.0% coverage and > 80.0% identity with FosC3 in the NCBI non-redundant database were all from *Aeromonas* spp. Most of them were found in *A. caviae* (31/54, 57.4%), and the others were from *Aeromonas* spp. (16/54, 29.6%), *A. dhakensis* (4/54, 7.4%), *A. bivalvium* (2/54, 3.7%) and *A. enteropelogenes* (1/54, 1.9%), respectively (Supplementary Table S1).

**Figure 4:**
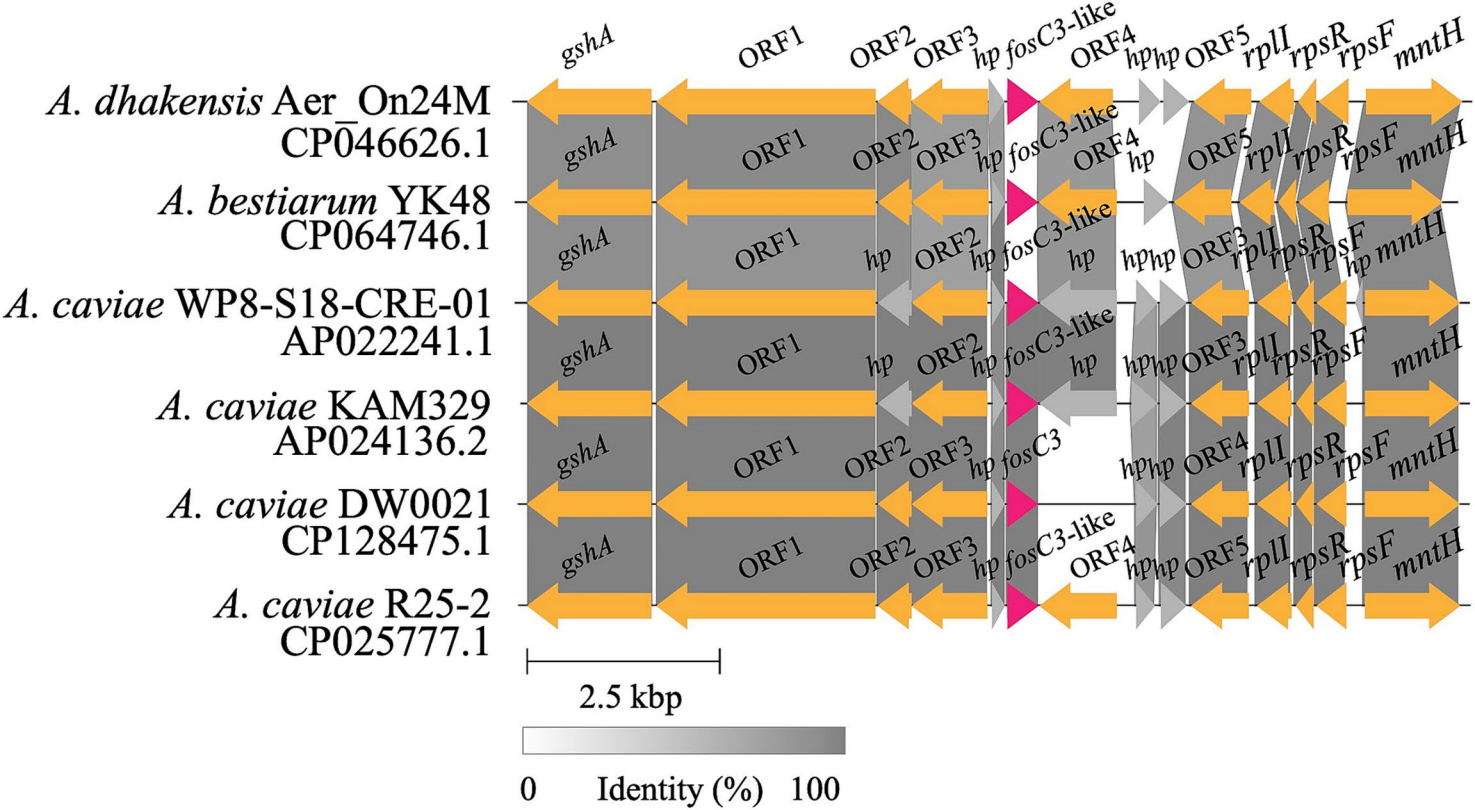
Alignments of the *fosC3* and *fosC3*-like genes clusters. A grey linked region indicated a ≥ 80% identity. *fosC3* and *fosC3*-like genes were highlighted in red. Genes annotated with ORF were those without official gene symbols. *hp*, hypothetical genes.

The *fosC3* and *fosC3*-like genes were located within a conserved genomic region (Figure 4). No mobile genetic element was identified around the flanking regions of *fosC3* and *fosC3*-like genes (> 80% identity). *fosC3* was surrounded by upstream genes encoding FxsA family protein, aspartate ammonia-lyase, and anaerobic C4-dicarboxylate transporter, and downstream genes of *fosC3* were ribosomal protein and so on. The structure of the *fosC3*-related fragment was similar to several other chromosomal fragments of *A. caviae* strains. The result indicated that *fosC3* may be a gene in a conserved genomic region of *A. caviae* strains. *fosC2*^AS^ was also encoded in a region without MGE (Ortiz de la Rosa et al., 2022). However, other *fos* genes such as *fosC2* and *fosA* genes were found related with the MGEs. The *fosC2* gene was found in a class 1 integron accompanied by *dfrA17* and *aadA5* encoded in a plasmid (Wachino et al., 2010). *fosA* was first discovered on Tn*2921* in a plasmid (Navas et al., 1990). Other plasmid-borne *fosA* genes such as *fosA3* (Wachino et al., 2010), *fosA5* (Ma et al., 2015), *fosA6* (Guo et al., 2016) and *fosA8* (Poirel et al., 2019) were also identified.

## Conclusion

A novel gene *fosC3* that encoding fosfomycin thiol transferases was identified on the chromosome of *A. caviae* DW0021, and the kinetic and functional properties of the FosC3 enzyme were measured. FosC3 shared the highest amino acid similarity of 58.65% (99.25% coverage and 59.09% identity) with FosC2 and phylogenetically related to FosC2 and FosG. FosC3 was able to inactive fosfomycin with a catalytic efficiency (*k*_cat_/*K*_m_) of (1.2 ± 0.3) × 10^3^ μM^−1^·s^−1^. This work contributed to the study on mechanism of fosfomycin resistance in pathogenic *Aeromonas* species such as *A. caviae*.

## Data Availability

The datasets presented in this study can be found in online repositories. The names of the repository/repositories and accession number(s) can be found in the article/Supplementary material.

## Footnotes

^1^ https://www.premierbiosoft.com/primerdesign/index.html

^2^ https://www.snapgene.com/

^3^ https://github.com/tseemann/barrnap

^4^ https://ftp.ncbi.nlm.nih.gov/entrez/entrezdirect/

## Supplementary material

SUPPLEMENTARY FIGURE S1 **(A)** Over-expression of enzyme. lane 1, expression without IPTG induction; 2, sediment; 3, supernatant; 4, supernatant (pCold I-*fosC3*/BL21); 5, sediment (pCold I-*fosC3*/BL21); M, 180 kDa marker. **(B)** Purification of enzyme. Lane M, 10-180 kDa marker; lane 1-4, EK digestion for 12 h, 8 h, 4 h, and 0 h under 4°C, respectively; lane 5, FosC3.

SUPPLEMENTARY FIGURE S2 HPLC chromatogram. Retention time (0–6 min): 3.32 min: unknown peak, 3.97 min: product peak, 4.68 min: GSH peak, 5.05 min: unknown peak.

SUPPLEMENTARY FIGURE S3 **(A)** Cartoon representation of the structure of FosC3 and **(B)** FosC3 bound with fosfomycin (residues 9T, 46W, 60Y, 88K, 92S, 98Y, 117R). **(C)** Metal coordination with FosC3. K^+^: 90N, 92S, 94G, 96S and Mn^2+^: 7H, 62H, 108E.
