# Supplementary figures and images for: Identification of a novel chromosome-encoded fosfomycin resistance gene fosC3 in Aeromonas caviae
Source: Front Microbiol. 2025 Apr 15;16:1577167. doi: 10.3389/fmicb.2025.1577167 (PMC12037509; doi:10.3389/fmicb.2025.1577167)

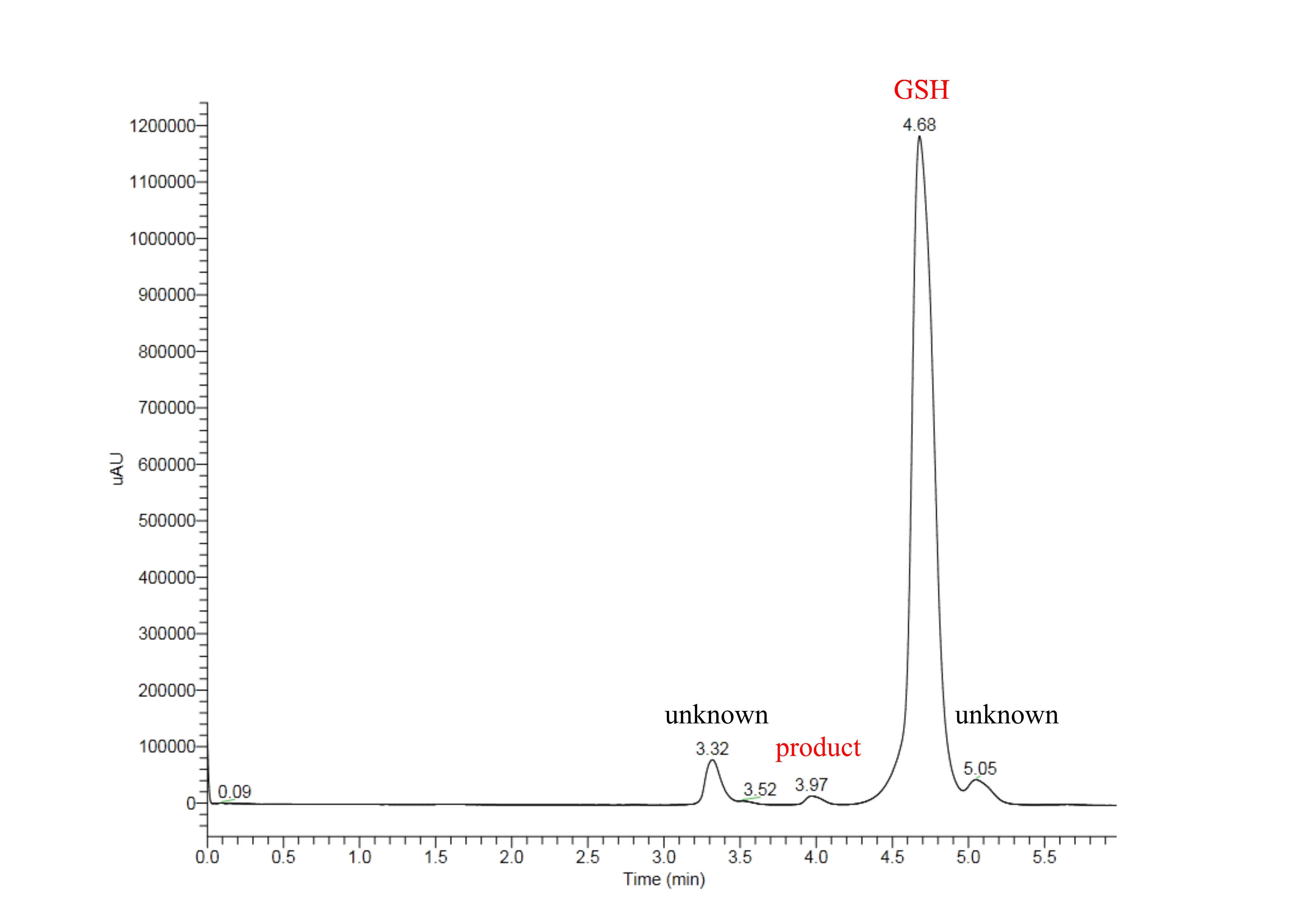

Supplement: SUPPLEMENTARY FIGURE S2 — HPLC chromatogram. Retention time (0–6 min): 3.32 min: unknown peak, 3.97 min: product peak, 4.68 min: GSH peak, 5.05 min: unknown peak. [file Image_2.jpeg]

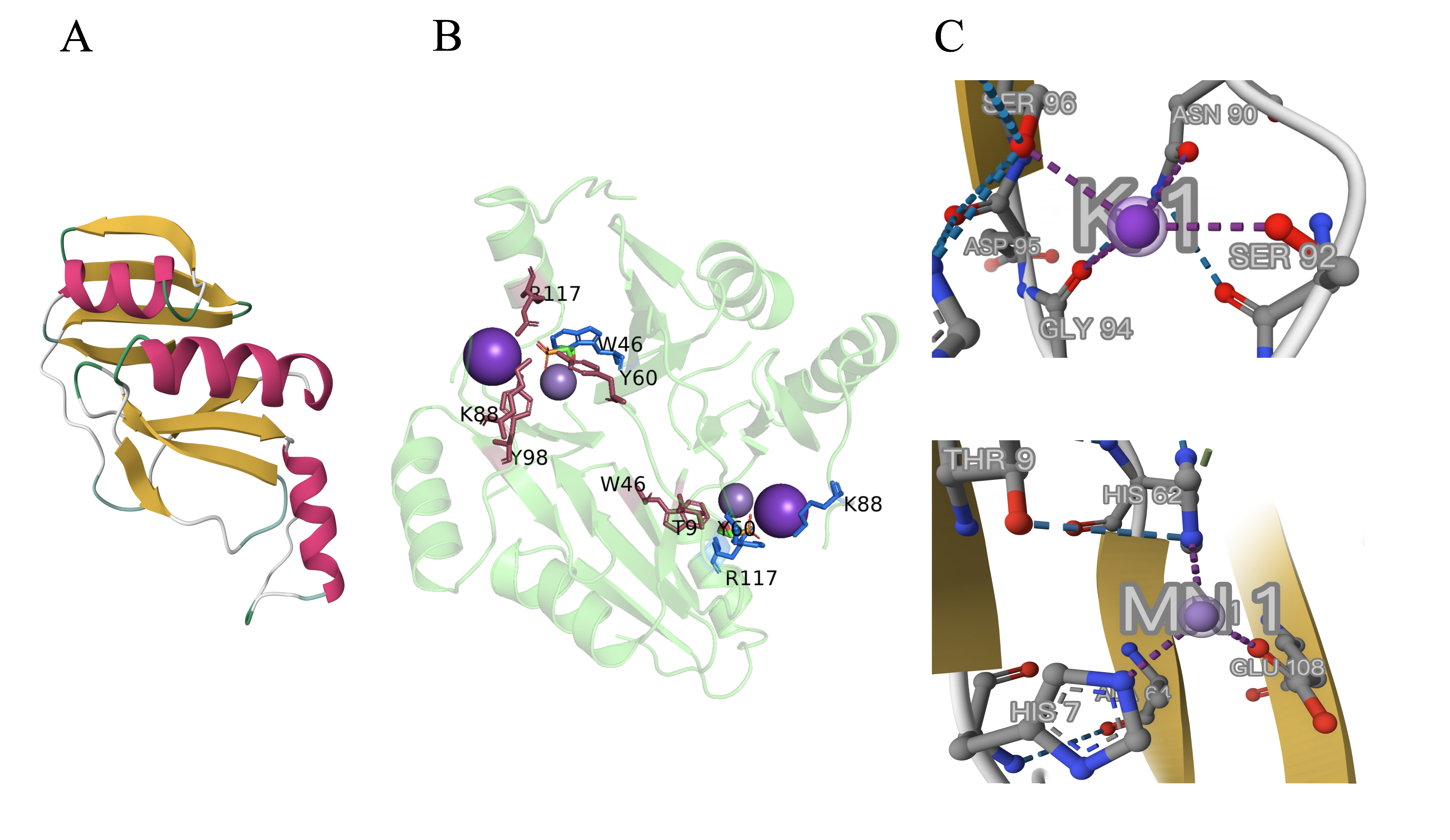

Supplement: SUPPLEMENTARY FIGURE S3 — (A) Cartoon representation of the structure of FosC3 and (B) FosC3 bound with fosfomycin (residues 9T, 46W, 60Y, 88K, 92S, 98Y, 117R). (C) Metal coordination with FosC3. K+: 90N, 92S, 94G, 96S and Mn2+: 7H, 62H, 108E. [file Image_3.jpeg]
