# Supplementary material for: Identification of a novel chromosome-encoded fosfomycin resistance gene fosC3 in Aeromonas caviae
Source: Front Microbiol. 2025 Apr 15;16:1577167. doi: 10.3389/fmicb.2025.1577167 (PMC12037509; doi:10.3389/fmicb.2025.1577167)
Supplement: Supplementary file 4 [file Table_1.docx]

**Table S 1. FosC3-like sequences in NCBI database.**

| Taxonomy | Query Coverage | E value | Identify (%) | Subject Length | Accession |
| --- | --- | --- | --- | --- | --- |
| *Aeromonas* sp. | 100% | 2.00E-92 | 100 | 133 | WP_039040005.1 |
| *Aeromonas* sp. Prich7-2 | 100% | 6.00E-92 | 99.25 | 133 | WP_210538001.1 |
| *Aeromonas caviae* | 100% | 6.00E-92 | 99.25 | 133 | WP_045523279.1 |
| *Aeromonas caviae* | 100% | 6.00E-92 | 99.25 | 133 | WP_113978528.1 |
| *Aeromonas caviae* | 100% | 6.00E-92 | 99.25 | 133 | WP_049636446.1 |
| *Aeromonas caviae* | 100% | 6.00E-92 | 99.25 | 133 | WP_128271556.1 |
| *Aeromonas* sp. QDB14 | 100% | 7.00E-92 | 99.25 | 133 | WP_270822040.1 |
| *Aeromonas caviae* | 100% | 7.00E-92 | 99.25 | 133 | WP_257710235.1 |
| *Aeromonas caviae* | 100% | 8.00E-92 | 99.25 | 133 | WP_128312886.1 |
| *Aeromonas* sp. | 100% | 9.00E-92 | 99.25 | 133 | WP_042864439.1 |
| *Aeromonas caviae* | 100% | 1.00E-91 | 99.25 | 133 | WP_048209162.1 |
| *Aeromonas* sp. | 100% | 1.00E-91 | 99.25 | 133 | WP_010673125.1 |
| *Aeromonas caviae* | 100% | 2.00E-91 | 99.25 | 133 | WP_279981135.1 |
| *Aeromonas* sp. | 100% | 2.00E-91 | 99.25 | 133 | WP_171864915.1 |
| *Aeromonas caviae* | 100% | 2.00E-91 | 99.25 | 133 | WP_257707503.1 |
| *Aeromonas* sp. | 100% | 3.00E-91 | 98.50 | 133 | WP_104451877.1 |
| *Aeromonas caviae* | 100% | 3.00E-91 | 98.50 | 133 | WP_223916272.1 |
| *Aeromonas caviae* | 100% | 3.00E-91 | 98.50 | 133 | WP_161470503.1 |
| *Aeromonas caviae* | 100% | 4.00E-91 | 98.50 | 133 | WP_261999175.1 |
| *Aeromonas caviae* | 100% | 4.00E-91 | 99.25 | 133 | WP_221365855.1 |
| *Aeromonas* sp. | 100% | 5.00E-91 | 98.50 | 133 | WP_160838886.1 |
| *Aeromonas caviae* | 100% | 6.00E-91 | 97.74 | 133 | WP_161646831.1 |
| *Aeromonas caviae* | 100% | 7.00E-91 | 98.50 | 133 | WP_041214493.1 |
| *Aeromonas caviae* | 100% | 7.00E-91 | 98.50 | 133 | WP_268451690.1 |
| Aeromonas sp. | 100% | 9.00E-91 | 98.50 | 133 | WP_109111794.1 |
| Aeromonas sp. | 100% | 9.00E-91 | 98.50 | 133 | WP_216944724.1 |
| *Aeromonas caviae* | 100% | 1.00E-90 | 98.50 | 133 | WP_139700527.1 |
| *Aeromonas caviae* | 100% | 2.00E-90 | 97.74 | 133 | WP_279990104.1 |
| *Aeromonas caviae* | 100% | 2.00E-90 | 97.74 | 133 | WP_257711836.1 |
| *Aeromonas caviae* | 100% | 2.00E-90 | 98.50 | 133 | WP_201888061.1 |
| *Aeromonas caviae* | 100% | 2.00E-90 | 98.50 | 133 | WP_041210974.1 |
| *Aeromonas caviae* | 100% | 2.00E-90 | 98.50 | 133 | WP_214003869.1 |
| *Aeromonas caviae* | 100% | 2.00E-90 | 97.74 | 133 | WP_201969738.1 |
| *Aeromonas* sp. LsrichE-8G | 100% | 2.00E-90 | 97.74 | 133 | WP_244812573.1 |
| *Aeromonas* sp. QDB12 | 100% | 3.00E-90 | 97.74 | 133 | WP_270667731.1 |
| *Aeromonas caviae* | 100% | 3.00E-90 | 97.74 | 133 | WP_201926778.1 |
| *Aeromonas* sp. QDB68 | 100% | 3.00E-90 | 96.99 | 133 | WP_270825116.1 |
| unclassified *Aeromonas* | 100% | 4.00E-90 | 97.74 | 133 | WP_270662299.1 |
| *Aeromonas caviae* | 100% | 5.00E-90 | 97.74 | 133 | QSO21479.1 |
| *Aeromonas* sp. | 100% | 1.00E-89 | 97.74 | 133 | MBP8281211.1 |
| *Aeromonas caviae* | 100% | 1.00E-89 | 97.74 | 133 | WP_203764156.1 |
| *Aeromonas caviae* | 100% | 2.00E-89 | 96.99 | 133 | WP_167825819.1 |
| *Aeromonas caviae* | 100% | 2.00E-89 | 97.74 | 133 | WP_223932072.1 |
| *Aeromonas caviae* | 100% | 2.00E-89 | 97.74 | 133 | WP_201935982.1 |
| *Aeromonas* sp. ASNIH8 | 100% | 3.00E-89 | 96.99 | 133 | WP_103858663.1 |
| *Aeromonas caviae* | 100% | 8.00E-89 | 96.99 | 133 | WP_102949181.1 |
| *Aeromonas caviae* | 96% | 3.00E-88 | 100 | 128 | WP_201872858.1 |
| *Aeromonas dhakensis* | 100% | 1.00E-86 | 93.23 | 133 | WP_065018286.1 |
| *Aeromonas dhakensis* | 100% | 3.00E-85 | 91.73 | 133 | WP_019839912.1 |
| *Aeromonas enteropelogenes* | 100% | 4.00E-85 | 90.98 | 133 | WP_017764028.1 |
| *Aeromonas dhakensis* | 100% | 7.00E-85 | 90.98 | 133 | WP_206219456.1 |
| *Aeromonas dhakensis* | 97% | 5.00E-83 | 92.31 | 130 | WP_208200086.1 |
| *Aeromonas bivalvium* | 100% | 4.00E-75 | 82.71 | 133 | WP_111874768.1 |
| *Aeromonas bivalvium* | 100% | 6.00E-75 | 81.95 | 133 | WP_041995071.1 |
